# Ontario Dairy Producers’ Perceived Barriers and Motivations to the Use of Pain Control for Disbudding and Dehorning Calves: A Qualitative Study

**DOI:** 10.3390/ani12080973

**Published:** 2022-04-09

**Authors:** Julia Saraceni, David L. Renaud, Erin Nelson, Jennifer M. C. Van Os, Cynthia Miltenburg, Charlotte B. Winder

**Affiliations:** 1 Department of Population Medicine, University of Guelph, Guelph, ON N1G 2W1, Canada; jsaracen@uoguelph.ca (J.S.); renaudd@uoguelph.ca (D.L.R.); 2 Department of Sociology and Anthropology, University of Guelph, Guelph, ON N1G 2W1, Canada; enelson@uoguelph.ca; 3 Department of Animal and Dairy Sciences, University of Wisconsin-Madison, Madison, WI 53706, USA; jvanos@wisc.edu; 4 Ontario Ministry of Agriculture, Food and Rural Affairs, Guelph, ON N1G 2W1, Canada; cynthia.miltenburg@ontario.ca

**Keywords:** welfare, dairy calf, analgesia, anesthesia, interview

## Abstract

**Simple Summary:**

This study aimed to understand influences on producer behaviour towards the use of pain mitigation for disbudding and dehorning. Calf comfort, post-operative performance, and better farmer experiences were common motivators for pain control use. Barriers included cost, education, and producer attitude. Quality assurance requirements for disbudding and dehorning practices were received well by participants; however, there were requests for more education surrounding the application of pain control for these procedures. Veterinarians were highly influential for participants and were mentioned as an avenue for the reduction of pain control barriers via producer education.

**Abstract:**

Canadian dairy farmers are required to use a local anesthetic and analgesic prior to all disbudding and dehorning procedures. This study was done to investigate the opinions of Ontario dairy farmers on the use of pain control for disbudding and dehorning calves and their perspectives on the current requirements of the quality assurance program. Interviews were conducted with 29 dairy farmers across Ontario. All participants used a cautery iron to disbud or dehorn their calves and some form of pain control (i.e., NSAID and/or local anesthetic). Of the 29 producers that were interviewed, 22 (76%) were in compliance with the proAction requirements for pain control. Many participants felt positive about the use of pain control for these practices. Education from veterinarians was one of the most commonly listed resources to reduce barriers to pain control use by producers. A farmer’s attitude was highly referenced as an influence on producer behaviour. Although participants had positive views of pain control use, full compliance with national quality assurance requirements for disbudding and dehorning was not met by all. Producer education through veterinarians is a potential avenue to encourage the adoption of pain control use for disbudding and dehorning practices.

## 1. Introduction

Disbudding (destruction of horn producing cells within the poll of young calves [1]) and dehorning (removal of the horn once it has formed attachment to the skull [2]) are common practices on dairy farms around the world. Disbudding and dehorning are done to reduce the risk of injury to animals and farm staff by horned cattle [3,4]. These practices are commonly used in Canada and the United States, with 96% of Canadian respondents disbudding or dehorning their calves [5] and 94% of US dairy operations reported having dehorned cattle [6]. 

All forms of disbudding (i.e., caustic paste, cautery iron) and dehorning (i.e., gouge, scoop, wire saw) are painful, as evidenced by behavioural, physiologic, and neuroendocrine changes during these procedures [3]. Use of a local anesthetic block, commonly a lidocaine injection, will virtually eliminate the acute pain response to all methods of disbudding and dehorning in calves [5,7,8,9]. In addition, systemic analgesia, such as non-steroidal anti-inflammatory drugs (NSAIDs), given at the time of disbudding and dehorning, reduces longer-term inflammatory pain associated with both procedures [3,8,9,10]. The combined use of a local anesthetic and systemic analgesic is recommended as the best practice for disbudding and dehorning by both the Canadian Veterinary Medical Association (CVMA) and the American Association of Bovine Practitioners (AABP) [2,11].

Dairy producers in Canada are required to follow standards from the quality assurance program *proAction*, which was developed by the Dairy Farmers of Canada and has had animal care requirements for producers in place since its initiation in September 2015 [12]. As of September 2019, the standards state that disbudding and dehorning must be performed with the use of a local anesthetic and an analgesic [13]. Use of pain control appears to have improved over time; the Canadian National Dairy study found that in 2015, local anesthetics, and NSAIDs were used by 66% and 25% of cautery users, respectively [5], which was higher than previous estimates from 2004, showing only 22% of Ontario producers using local anesthetics for these procedures [14]. Although substantial progress has been made, the real or perceived barriers that are preventing the full adoption of best practices are unclear. 

Qualitative research has often been used by researchers seeking to better understand influences on human behaviour [15,16,17]. Through this research approach, it is possible to identify the motivators and barriers that encourage and inhibit certain behavioural practices, such as the use of pain control for disbudding and dehorning. Understanding the motivations and barriers that influence producers’ behavioural decisions will help researchers identify potential routes of positive change to encourage the adoption of best disbudding and dehorning practices by producers.

The objective of this qualitative study was to gain an understanding of the motivating factors that encourage the use of pain control for disbudding and dehorning for a proportion of Ontario dairy producers, as well as the barriers that are preventing these farmers from complying with required practices. This study also investigates the opinions of 29 Ontario dairy producers regarding the current disbudding and dehorning requirements of proAction.

## 2. Materials and Methods

### 2.1. Ethical Approval

This manuscript is reported following the Consolidated Criteria for Reporting Qualitative Research (COREQ) 32-item checklist for interviews and focus groups [18]. This project was approved by the University of Guelph Research Ethics Board (no. 20-03-016). All participants of this study gave written or verbal consent before participation. 

### 2.2. Research Team and Reflexivity

All interviews were conducted and analyzed by one female researcher (JS). The research team hired an external transcriptionist to transcribe all recorded interviews. The primary researcher (JS) was an MSc candidate at the University of Guelph in the Department of Population Medicine and was supervised by a team of committee members throughout the duration of this study. JS has a particular interest in animal health, welfare, and agriculture, and has previous experience with conducting scientific research studies from the completion of her BSc degree in Biology. Prior to participation, study participants were informed of the study objectives and the goals of the primary researcher. No personal relationships were established with participants prior to study commencement. 

### 2.3. Theoretical Framework

This study focused on the interpretivist (or constructivist) research framework and utilized inductive reasoning for data analysis. This research paradigm makes use of relative and subjective ontological and epistemological views [19], allowing for differences in social behaviours to be explained through contextual differences between individuals [20]. The methodological orientation of this research did not follow one specific, established research tradition, but utilized more of a generic qualitative research approach, touching on aspects from different qualitative approaches. While aspects of deductive reasoning were touched on for data analysis, inductive reasoning allowed for hypothesis construction to be developed following thematic analysis and coding. Analysis and research strategies surrounding individuals’ lived experiences were inspired by the phenomenological research approach [21]. The development of the interview question guide was inspired by phenomenology. Internal lived experiences of participants were investigated through interview questions inquiring about participants’ farming experiences, their histories with disbudding or dehorning methods, and their on-farm relationships with staff, family members, and veterinarians. The phenomenological approach was useful in guiding the researcher in listening to and learning from the experiences of others for analysis purposes. 

### 2.4. Study Population and Participant Recruitment 

Phone interviews were conducted from August 2020 through January 2021. Study participants were recruited through advertisements placed in a producer magazine and on Twitter, as well as through industry contacts (veterinarians) and participant referral. Eligibility criteria of participants included English-speaking dairy producers within Ontario. Participants were required to be at least 18 years of age, able to read and understand the consent document that was provided to them prior to the interview, and able to understand and respond to interview questions about *proAction* and disbudding and dehorning practices. All Ontario dairy producers who were willing to participate in this study were interviewed and their responses analyzed. Analysis consisted of responses from a convenience sample of 29 participants. Sample size was based on that suggested for sole source interview research [22]. Participants received a CAD 25.00 gift card as compensation for their time and gratitude for their participation in the study. 

### 2.5. Study Design

A semi-structured discussion guide was created by the research team prior to the commencement of the study to ensure consistency between interviews and to guide the discussion with participants. The semi-structured nature of the guide allowed the researcher to ask consistent questions to all participants, while tailoring the specific language of questions to each participant to encourage a rapport between the participant and researcher. Introductory questions were included in the discussion guide to build rapport with the participant and provide demographic information to the interviewer. The discussion guide (Appendix A) was pilot tested by 2 individuals (1 dairy producer, 1 dairy farm employee) to ensure appropriate interview length and clarity of questions. One pilot interview was included in the final analysis since no substantial changes were made to the interview guide and the participant met the inclusion criteria to participate in the study. The interviews took approximately 30 min to complete over the phone, with no face-to-face interaction between the participant and interviewer. This was done to abide by COVID-19 safety protocols. The interviewer (JS) and the participant were the only individuals present during the interview. A research log was kept of written notes taken during all conversations with participants. All interviews were audio recorded using the MacBook Pro Voice Memos app (Version 2.0, Apple Inc., Cupertino, CA, USA). 

A brief statement was read to participants by the researcher at the start of the interview. This introduction reviewed participant consent and confidentiality, as well as the role and objectives of the interviewer. A total of 17 main questions and 23 prompting questions were presented to the study participants throughout the interview. Interview questions were based on pre-determined study objectives and covered 4 main topics of interest: participant demographics, current disbudding or dehorning protocol, pain medication for disbudding and dehorning, and *proAction* requirements. 

Demographic questions inquired about the size of the participant’s herd, the location of their farm, and the participant’s experience with dairy farming. Participants were then asked about the details of their current disbudding or dehorning protocol, including calf age, medications provided, disbudding or dehorning method, protocol development, who performs the disbudding and dehorning on their farm, and how long they had been using their current disbudding or dehorning protocol. Questions were asked regarding participants’ general opinions on analgesia and anesthesia for disbudding and dehorning, as well as how they felt other members of the dairy community perceived the use of pain control for these practices. 

Motivations for and barriers against the use of pain control were discussed, with participants also being asked to give their perspectives on how other members of the dairy community might feel about the use of pain control for disbudding. Participants were also asked to speak on how they felt barriers to pain control use might be eliminated or reduced, and if they felt that pain mitigation should be provided for disbudding and dehorning regardless of the real or perceived barriers that producers are currently facing. Lastly, participants were asked their opinions on the current *proAction* requirements for disbudding and dehorning. As a closing question, participants were asked if they wanted to share any final thoughts or discuss any topics that had not been previously mentioned in the interview. 

Repeated interviews were not conducted with participants. Participants were offered the opportunity to receive a copy of the transcribed interview if they so desired and provide any feedback to the researcher on the interview process. Participants were given the opportunity to review their interview transcript, at which point they would have had the opportunity to remove content from the transcript if desired. No participants requested to review the transcript following their interview. While data saturation (reaching a point in data collection when no new responses are obtained; [23]) was reached with many topics and questions discussed in the interview guide, interviews were carried out past the point of saturation to allow all interested individuals the opportunity to participate. Throughout this manuscript, square brackets are used within quotations from participants to provide context where missing. 

### 2.6. Thematic Analysis

All transcribed interviews were analyzed by one researcher (JS) and coded using NVivo 12 software (version 12.6.0, QSR International, Doncaster, Australia, 1999). Interview transcripts were compared to audio files for accuracy. A single researcher (JS) coded all transcripts. All data were analyzed using thematic analysis as described by Aurini et al. (2016). The author of this research read through the transcripts repeatedly to fully immerse herself in the data (i.e., the researcher developed a deep understanding of the data prior to analysis). Broad themes and general topics were identified before data collection, while more specific themes were derived from the data once the analysis began. A coding tree was used to identify themes within the data, starting with more high-level, broad sections, and then narrowing the scope of the coding lens (a coding lens refers to the scope of interpretation that the researcher uses during analysis [24]) to identify more specific topics and themes. The interview questions and their subsequent responses were organized into 4 broad sections based on the previously mentioned topics of interest (respondent demographics, disbudding and dehorning protocol, pain control for disbudding and dehorning, and *proAction* requirements). Within each of these main topics, smaller sub-sections were created that highlighted overarching themes within the questions being asked (e.g., ‘personal view of pain control’ as a sub-section within the ‘pain control’ topic). A third, more specific level of coding was used for the responses to each question. Responses were coded by highlighting themes that emerged from the text and organizing them into third-tier sections within the pre-existing sub-sections (e.g., ‘better calf recovery’ as a coded section within the sub-section ‘personal view of pain control’). The full data set was coded line-by-line as an initial form of analysis. As new codes were created at each level, a codebook was created to describe and define each code. Once the codes had been redefined, the data were recoded one final time using the redefined codebook. The coded sections of the data were then organized into a text document and summarized to be expanded and then collapsed into major themes and topics present within the text. These themes were analyzed to ensure that they accurately reflected the dataset, as well as to identify any connections between topics or overarching key ideas present in the data (i.e., the key topics that were present throughout the data that reflected the major ideas present within the data [23]). Selected quotes from various individual participants have been included throughout the results section to provide the reader with greater context. The quotes presented were carefully selected by the researcher (JS) after in-depth coding, thematic analysis, and additional review. The selected quotes are statements that researchers feel best represent the themes presented in this manuscript. These participant statements are highly meaningful, and their inclusion aims to support the reader in better understanding the perspectives of the dairy producers within this study.

## 3. Results

### 3.1. Respondent Demographics

A total of 29 dairy producers were interviewed across Ontario, 22 of which were male and 7 were female. Farms were located throughout Ontario as far south and west as Troy, as far north as Westmeath, and as far east as Moose Creek. The mean (±SD) farm size was 124 ± 113 milking cows, with a range of 27 to 500 cows. Of the participants who described the staff available on their farm (*n* = 9), one farmer mentioned having a well-established staff; however, many (*n* = 8) were family run with few to no employees. Many (*n* = 12) producers were generational farmers, with parents and grandparents having been involved in the industry prior. Producers’ personal experiences with dairy farming ranged from less than one year to over 30 years in the industry.

### 3.2. Disbudding and Dehorning Practices

Participants reported disbudding and dehorning calves as young as 2–3 days old, with the oldest at up to 5 months. All producers reported using a cautery iron. Two respondents also mentioned using gougers to dehorn calves that were overlooked and had grown horns too large to be removed via cautery. Most respondents indicated that they were the primary individual who performed the disbudding or dehorning on their farm. For additional context, Table 1 describes the disbudding and dehorning practices of interview participants.

### 3.3. Disbudding and Dehorning Protocol

Producers stated that they had been using their current disbudding or dehorning protocol for quite some time, ranging from less than one year to 45 years using their preferred protocol. A few producers mentioned using their current protocol for many years but incorporated meloxicam when it was made available to them, or since the time that *proAction* required the use of an NSAID for disbudding and dehorning.

Producers often mentioned that their current disbudding or dehorning protocol was developed over time as they looked for ways to improve their current protocol through trial and error. Protocol development also came from being informed about a better disbudding or dehorning method through other producers, newly publicized information, and their veterinarian, with veterinarian being the most commonly reported influence for producers on their current disbudding or dehorning protocol. The public was mentioned as an influential factor, with producers reporting their methods as being more pleasing to the public: “from the public eye, and we do tend to be in the public eye a lot, I think that it just looks a little better when we are doing everything to make this a…less painful experience”.

### 3.4. Disbudding and Dehorning Medications

All producers that were interviewed in this study used some form of pain control medication (i.e., anesthetic and/or analgesic) when disbudding or dehorning their calves. While some participants used only one of the two required pain control medications for disbudding and dehorning (i.e., either an NSAID or a local anesthetic), the majority of participants were in compliance with the *proAction* requirement for disbudding and dehorning that states the mandatory use of both an NSAID and a local anesthetic. For additional context, Table 2 describes a further breakdown of the medications used by interview participants. A few producers mentioned issues with administering medications that prevented them from being in compliance with the disbudding and dehorning requirements from *proAction*, such as difficulty performing the lidocaine local nerve block and difficulty with dosing and timing of administration when using a lidocaine solution that was mixed with a sedative (typically xylazine; this is also supported by [25], which highlights xylazine as being commonly used for sedation): “[lidocaine block] just didn’t work for us so that’s why on a daily basis when we do dehorn we’re not actually using the freezing. We’re just using the [meloxicam]”.

### 3.5. Dairy Farmer Opinions on Pain Control

#### 3.5.1. Participant View of Pain Control Medication

Participants were asked to describe how they felt about the use of pain control medications for disbudding and dehorning. Often, interview participants felt that pain mitigation was necessary for disbudding and dehorning, stating that it provided a better experience for the calves. When asked what the main motivations towards using pain control for disbudding and dehorning would be, participants overwhelmingly reported calf comfort and better post-operative recovery as the most common responses. Participants felt that using pain control was a more humane method of disbudding and dehorning, describing their passion for farming and concern for their animals as motivations towards using pain control for these procedures: “I think most of us are just fairly concerned about the wellbeing of our livestock. So if we can do something that improves that, [or] minimizes any negative impacts of practices that we have to carry out I think that’s a bigger motivator than any regulation”. Producers felt that they had a responsibility to their animals to treat them in the most humane way possible and often compared disbudding and dehorning calves without pain control to having a dental procedure performed without pain mitigation: “my thoughts are [you] wouldn’t go to the dentist and have a tooth pulled out without having it frozen. I wouldn’t …it goes hand in hand [with] disbudding”. Interview participants also felt that using pain mitigation for disbudding and dehorning creates a better experience for the producer, stating that it was inexpensive, helpful, allowed the producer to do a better job of the procedure, and made the process easier, safer, and faster.

A few participants discussed the cost associated with disbudding and dehorning medications, stating that the benefit that is received from using pain control largely outweighs the cost and that it is a worthwhile investment. As one participant stated: “it just helps the calf to keep growing so [it’s] beyond just animal welfare. It’s also economical to give them that and then have them keep growing better than to set [them] back right?”. Some producers recognized that although using pain control was helpful, it increased the time and cost associated with the procedure: “The unfortunate part about meloxicam is I do think it’s a little bit on the pricey end of things…it’s really hard to kind of quantify the gains that you would establish there”.

Another commonly mentioned motivation towards using pain control for disbudding was education of producers via influential sources (i.e., published research, veterinarians). Participants felt that once they had been educated on the benefits of using pain control, it largely motivated them to uptake this practice. Disbudding and dehorning requirements for producers and consumer perception were also listed as motivating factors towards the use of pain control, with participants stating concerns of how the public would view disbudding and dehorning without pain mitigation: “I think for consumers too like to say hey, you know um, one they don’t probably even like you to-taking the horns off the animals, but once you explain why they usually understand, but then if you say hey, I don’t use any pain medications they’re like what are they gonna think?”.

Some producers felt that pain control was not always necessary for disbudding and dehorning and that the use of either NSAIDs or local anesthetics was dependent on the calf and the situation. One participant said: “it all depends on the calf. Because sometimes the way we do it is sometimes the calves don’t even know. Like they don’t even seem to care. But you get the other ones…that seem to care a lot”. Another participant had a similar comment: “You know if you look at my process I use a bit of pain control, but I use it after the fact right? So… if you’re doing the animals when [they’re] young and small…in my opinion you may not need pain control”. A few producers mentioned not utilizing lidocaine for disbudding and dehorning because of hesitancy with administering the local anesthetic block: “I haven’t started freezing them yet because…I’m nervous of sticking that needle in behind [the eye]”. One producer felt that the needle application in the cornual groove was stressful for the calves and might be more painful than the brief pain associated with disbudding without pain control: “did anybody do any research…to determine how much pain the animal experiences when you inject the lidocaine? Because that’s got to hurt when you put a needle…to deliver the lidocaine”.

#### 3.5.2. Perceptions of Other Producers’ View of Pain Control Medication

Participants were asked to describe their views of what other dairy producers (i.e., neighbours, colleagues, farmers in their community, etc.) thought about the use of pain control medication for disbudding or dehorning. Interview participants thought that other dairy producers would view pain control as beneficial to the calf and to the farmer, and that they would be concerned for the welfare of the animal. Many participants felt that other producers had a positive view of pain control for disbudding and dehorning because they felt that it made the process easier, less painful for the calf, and provided better recovery in the days following the procedure. Of the farmers that they regularly associated with, participants stated that most used pain control for disbudding or dehorning and that they viewed it to be important: “lots of people I do talk to or have talked about it yeah, they do like it too. They see a difference for sure, yeah”. Veterinarians were thought to be an influential source, with a few producers stating that other dairy producers would be more likely to use, and be educated on, pain control if their veterinarian performed the disbudding or dehorning on the farm. Some producers also mentioned public perception as an instigator for making positive change and something that other producers were also conscientious of: “I think consumers already have a negative enough vision of agriculture that we don’t need to go out of our way to make ourselves look bad. We should be doing things collectively to say hey, we’re doing the best things we could do right now”.

When asked, participants felt that overall, other members of the dairy community had a positive view of pain control use for disbudding and dehorning; however, more of a mixed opinion on the subject was also represented, with participants indicating that the dairy community was fairly divided in terms of those who used pain control and those who did not. One participant said: “I think that probably we’re pretty divided as a community as to whether or not it should be required. Farmers in general don’t like being required to do things, right?”. Participants who did not comment on other dairy producers’ perspectives were unsure of how their peers would feel about pain mitigation for disbudding and dehorning and mentioned not discussing the topic with other producers. Participants felt that the views of other producers would be dependent on their education on the topic, mentioning that if a producer is educated on the benefits of pain control for disbudding and dehorning, they would be more likely to use it and view it as a positive tool.

Throughout the interviews, participants often mentioned the idea that the dairy community was divided into progressive farmers and ‘old school’ farmers. Participants described a generational feel to the current farming community, with calf care practices often being dependent on the age or generation of the farmer: “There are a lot more young people. They’re a lot more progressive. They’re a lot more in tune with…what’s required for good animal husbandry I feel”. Some participants felt that older producers were unwilling to change their ways of farming and might be more resistant to there being requirements for farmers, while younger and more progressive farmers are more interested in adapting to new changes in the industry: “I feel that progressive farmers seem to have the same opinion as myself…But…I don’t know how to describe them, like old dude farmers? Seem to resent what [*proAction*] tells them to do, and don’t seem to see the advantage as much”. When asked to describe what a progressive farmer looks like, one participant said: “people who read articles, and try to apply it? So people who try to always improve what’s happening on the farm…usually farms who have kids who are going to take over the farm, or younger farmers”.

#### 3.5.3. Barriers against Pain Control Use

All the participants in this study used at least one form of pain control when disbudding or dehorning. However, when asked what factors might prevent producers from using pain control for these practices in general, most participants had opinions on the topic or were able to speculate about what might be deterrents for other farmers. Most mentioned cost as a major deterrent for the producers that are not already using pain control. One participant said: “I think cost is probably gonna be your biggest sticking point”, while another mentioned that “[other producers] just think it’s cheaper and easier just to burn them off and be done with it…rather than looking at the overall health of the calf. They’re just looking to the convenience of just doing it and saving the money”.

Education was also commonly mentioned as a barrier to pain control use, with participants stating that producers who were not informed of the benefits or trained in administering the medications would not use pain mitigation. Some producers mentioned that administering the lidocaine block would deter other producers from using pain control because it is an intimidating process: “I’m not sure how many guys have actually you know, sat down and been shown [by] a vet, or by another farmer or someone with some experience how to find that nerve, how to block it properly and that kind of thing. So they just kind of keep going the way they’ve always gone”. A few participants stated that that they themselves were hesitant to administer lidocaine for this same reason.

Through this study, we were able to further investigate a small proportion of Ontario dairy producers’ opinions on the use of pain mitigation and identify avenues to eliminate barriers to pain control use for disbudding and dehorning. Participants felt that producers’ attitudes are influential on their decision to use pain control and the producers who are unwilling to change old practices and view their animals strictly as a source of income, would not be inclined to use pain control because they do not feel it is necessary: “[Some farmers still] look at animals in a purely…means to an end…They’re looking at it as more like [a] tool…so yeah, that might be the reason…‘that’s the way we’ve always done it’. There’s always that reason…not aware…not open to change”. Producers’ attitudes towards calves may influence their perception of the necessity of pain mitigation. In addition, it has been highlighted by participants that producers with poor attitudes towards making behavioural changes would be less inclined to change old practices to adopt pain control use. One participant said: “I don’t think there are any barriers…I think the barrier is in the producers themselves”. Some participants reflected on the ability of the producer to have some level of preparedness and organization for these procedures, indicating that administering pain control may be viewed as an inconvenience or time consuming if producers do not consider it a priority to get the medications ahead of time.

#### 3.5.4. Removal of Barriers

When asked how barriers against the use of pain control for disbudding and dehorning could be removed for producers, participants most frequently mentioned utilizing veterinarians as an educational influence. Participants felt that having a veterinarian administer medications for disbudding and dehorning, and/or having veterinarians educate producers on how to administer pain mitigation and why it is so beneficial, would be the best way to encourage the adoption of this practice: “Well your vet’s your best contact…a large percentage of …farmers in general are going to have a good relationship, or a working relationship at least with their vet. And so that would be your [best] avenue there”. Veterinarians were often mentioned as educational, influential, and trusted sources for participants, along with company representatives, other producers, community groups of farmers, and novel research: “I think it also needs to be producer driven…it’s one thing to have a veterinarian come up or someone who’s involved with the *proAction* directly, but if you’re the farmer actually on the receiving end of that I think you-your fellow producers need to come up and say hey, this is what we do on our farm and-and this is what really works”.

Some participants mentioned the use of government programs and requirements as a way to encourage the use of pain control, with discussion of implementing more enforcement for the programs that are already in place. One participant also mentioned creating incentives for producers as a way to encourage pain control use. A few producers stated that the use of polled genetics would be beneficial in eliminating barriers to pain control use and that incorporating more polled genetics into dairy farming was a positive direction for the industry; however, it was stated that there is still work to be done before polled genetics meet the standards of the dairy industry and it will take some time before majority polled herds are prominent in dairy farming.

### 3.6. proAction Disbudding and Dehorning Requirements

Most participants had positive responses when asked about the disbudding and dehorning requirements from *proAction*, regardless of their compliance with the program. Participants felt that the requirements were fair, easy to implement, good for public perception, made disbudding and dehorning easier, allowed for standardization of practices, and saved time. Many participants stated that they had already been using pain control before the disbudding and dehorning requirements were made mandatory.

A few participants felt that the disbudding and dehorning requirements were not always necessary and thought that they should be dependent on other factors. These participants mentioned that the requirements were beneficial as they encouraged the use of pain control for disbudding and dehorning; however, they felt that the requirements were not necessary for them or their calves specifically, based on how they perform the procedure or the age of their calves. One participant said: “I also think based on our experience it’s a huge difference whether you do them at 3 or 4 weeks of age or whether you do them at 10 or 12 weeks of age…I find those three seconds per horn of initial pain doesn’t really justify the hassle…when I just feel the [meloxicam] in our situation…is more beneficial to the animal than also freezing it”.

Some modifications to the requirements were suggested, such as including education or training on how to administer the local anesthetic block, allowing for a different medication timeline (i.e., allowing meloxicam to be administered after the procedure), flexibility with medication requirements, including sedation as a requirement, and better enforcement of the requirements. Overall, participants had mixed opinions regarding how other members of the dairy community felt about the disbudding and dehorning requirements from *proAction*. Some participants felt that many producers were already using pain control and received the requirements well, while others felt that producers do not adapt well to change and resent being told what to do, particularly the older generation of farmers.

## 4. Discussion

All participants used pain control for disbudding and dehorning, and many stated animal welfare concerns as a motivation for this. A farmer’s attitude, which refers to the general attitude and perspective of the producer as determined by many pre-existing factors such as age, environment, and personal experiences, was considered to be influential on behaviour. Barriers against pain control use were cost, education, and farmer attitudes. In general, the *proAction* requirements for disbudding and dehorning were seen to be positive for the dairy industry; however, further information and training on the administration of pain control medications is needed. This study summarizes the opinions of 29 dairy producers in Ontario and is not a direct reflection of the perspectives of a wider population of Canadian dairy producers.

### 4.1. Respondent Demographics

The average number of milking cows for participants was 124 ± 113 (mean ± standard deviation). This is larger than the provincial average of 95 cows [26], which indicates that this sample of participants may not be entirely reflective of the provincial dairy production population; however, the goal of qualitative research is not to find consensus in the data, or be representative of, or generalized to, a larger population [19], but rather to provide further depth from individual perspectives and behaviours [20]. Many participants mentioned that their farm was family-owned and operated, which is not surprising, as 98% of Canadian dairy farms are of the same structure [27].

### 4.2. Farmer Attitudes

The findings of this research indicate that participants’ attitudes towards pain control are influential on their decisions to use pain mitigation when disbudding or dehorning their calves. Farmer attitudes in general, appear to result from, and reflect, many factors such as producer attitudes towards changing behaviour and adopting new practices, farmers’ perception of their calves, generational differences, and previous experiences. ‘Farmer attitude’ as a general term can be used to encompass a producers’ perception in general, as influenced by a multitude of factors. Attitudes and perceptions have been shown to influence producers’ behaviour and decisions in a number of capacities, including in regard to measures of animal welfare. Studies of farmers in Norway found that producers who had higher indicators of empathy towards animals had the lowest prevalence of skin lesions in their herds [28]. This indicates an association between certain on farm animal welfare indicators and producer attitude towards animal pain.

Although all participants in this study used some form of pain mitigation for disbudding and dehorning, the producers in this study often mentioned farmer attitudes as a barrier towards other producers’ use of pain control. Participants often stated that other producers who do not use pain control might do so because they are unwilling to change their old habits and view their cattle as strictly a source of income. If producers tend to look at their calves as a means of income rather than an animal under their care, they may be more likely to prioritize other factors, such as money, time, or convenience, over the wellbeing of the calf. Wikman et al. [29] found that producers who took disbudding pain more seriously and felt that disbudding calves without pain mitigation would be painful for the animal, had a higher sensitivity to pain caused by cattle diseases. These results highlight the importance of farmer attitudes and beliefs as influences on how they view animal pain and welfare. Producers with a more animal-centered view of the dairy industry were considered to be “progressive” farmers by the participants in this study. Changing the attitudes of “old school” producers to view dairy farming in more of a progressive light and make animal welfare a priority, has the potential to increase the adoption of pain control use for disbudding and dehorning, thereby positively impacting the dairy industry going forward.

Social science theories have taken a deeper look at the factors that are involved in changing behaviour and how an individual’s attitude directly impacts this change. Livestock research and epidemiological studies have used the theory of planned behaviour (TPB) and the transtheoretical model (TTM) to understand patterns of decision making and behaviour of producers and their on-farm practices. The TPB argues that the likelihood of an individual to perform a certain action is dependent on their intention to perform that behaviour, and an individual’s intention or willingness to perform certain behaviours is dependent on their attitude, their subjective norms, and their perceived behavioural control [30]. The TTM presents a similar model, with individuals being identified as agents of change in their own lives; however, the individual’s mindset and expectations are seen as pre-conditions that affect the effectiveness of this change [31]. These theories explain the direct link between producers’ attitudes and perspectives, and their decisions to participate in specific on-farm practices, such as the use of pain control for disbudding and dehorning. A producer’s belief that the welfare of their animals is a greater priority than other factors (i.e., cost) can encourage the use of pain control for disbudding and dehorning, as well as the implementation of other calf-care practices that improve animal welfare. Encouraging a change in the attitude of producers who are more inclined to prioritize other aspects of dairy farming over calf welfare makes it possible to change the patterns of behaviour of this group. The connection between producer attitudes and decisions to enact change and patterns of behaviour, highlights the importance of producers’ opinions on the use of pain control and how these beliefs impact the adoption of this best practice.

### 4.3. Barriers to Pain Control Use

Participants mentioned farmer attitudes, cost, and lack of education about the procedure as primary barriers that prevent other producers from using pain control for disbudding and dehorning. Participants often stated that if other producers were educated on the benefit of pain control for disbudding and dehorning, they would be more inclined to adopt this practice. Evidence suggests that in addition to reducing the pain associated with disbudding and dehorning, use of analgesics can improve rumination initiation times and increase average daily gain for post-operative calves [3,32]. Cardoso et al. [17] found that producer education was also a significant barrier to pain control use with Brazilian farmers indicating that they did not use pain control for disbudding and dehorning because they were uneducated on the medications and/or did not think it was necessary for the procedure. The producers in this study felt that the use of pain control was not necessary because animal suffering is brief and “young” calves (producers identified young calves as calves up to 8 months of age) felt less pain during the procedure. Understanding how lack of education on pain mitigation presents a significant barrier for the use of pain control for disbudding and dehorning by producers, provides an opportunity to eliminate this barrier through the improvement of farmer education on this practice. By utilizing veterinarians and other educational resources, producers can receive more targeted education on the efficacy, necessity, and importance of using pain control for disbudding and dehorning, as well as better training on the application of pain mitigation to reduce hesitancy and increase adoption of pain control use.

Cost was also frequently mentioned as a barrier to pain control use by other producers. This supports previous research by Gottardo et al. [33], which mentions cost of analgesia as the primary barrier to pain control use for disbudding and dehorning by producers. Participants in this study mentioned that producers who do not view the cost of pain control medication as an investment in the future production of their cattle will be discouraged by the upfront cost of the medication. Education could be an avenue to change the way producers view this expense and to change the narrative surrounding the cost associated with welfare practices; however, this brings to light a new topic of conversation in the responsibility of farmers to support the welfare of their animals, regardless of the cost to the producer.

Participants mentioned the division in the dairy community between “progressive” farmers and “old school” farmers and the mixed opinions on the use of pain control for disbudding and dehorning through this division. Participants felt that progressive farmers who view animal welfare as a priority are not concerned by the cost associated with pain control use and feel a responsibility to put the welfare of their animals above all other things, including cost. A survey of Wisconsin producers may support this theory, with younger participants (aged 18–34) being more likely to have made changes to their disbudding or dehorning protocol in the last decade when compared to older participants (35–54) [34]. However, older producers in other research were seen to view cattle diseases [28] and horn removal procedures [29] as more painful than younger producers. This indicates that the divide between “progressive” and “old school” farmers may not be related to generation or age, but rather producer opinion and view of the dairy industry in general. The idea of a “progressive” farmer may not necessarily be linked to a younger demographic; however, understanding this contrast between producers sheds light on the disconnect that the dairy community is currently facing and highlights the potential for the future of this industry. By identifying barriers to pain control use as being related to farmer attitudes, a continual increase in more progressive-minded farmers through increased education of this demographic shows promise for improvement in animal welfare and uptake of best calf care practices by producers.

### 4.4. Producer Influences as a Method to Remove Barriers Surrounding Pain Mitigation

Veterinarians were highly regarded by participants and frequently mentioned as a tool to remove barriers against pain control use for other producers. Previous research highlights the importance of veterinarians as an influential source on producers in adopting disease prevention and biosecurity measures on their farms [35,36,37]. In terms of disbudding and dehorning practices, veterinarians were also seen to influence the use of anesthesia, analgesia, and change of disbudding or dehorning practices [25,38]. In addition, a study of Wisconsin dairy producers revealed that participants were more likely to use pain control for disbudding and dehorning if their veterinarian was involved in creating the protocol for these practices [34]. Veterinarians seem to be aware of the influence they have on producer behaviour and attitudes, with focus groups revealing that Canadian veterinarians feel a responsibility to shift normative attitudes towards calf-care practices and improve calf welfare through education of clients and social influence [39]. Veterinarians have an opportunity to discuss key animal welfare topics with their clients, highlighting not only the importance of pain mitigation for painful calf procedures, but also the importance of calf management in general. From the results of this study, we can infer that veterinarian education through informed, shared decision-making can be an effective tool to reduce barriers and encourage the uptake of best disbudding and dehorning practices. The influence of veterinarians as an educational tool for producers is potentially the most effective solution to reducing barriers to pain control use for disbudding and dehorning by producers.

Consumers and the perception of the public were also frequently mentioned as influential stakeholders on the practices of Ontario dairy producers. Similar concerns were identified by Wisconsin producers, who listed veterinarians and the public as the two most frequently selected influences on producers’ decision to change their pain control medication in the last decade [34]. Consumers are considered important industry stakeholders by producers in Great Britain as well, with Brennan et al. (2016) and Richens et al. (2018) identifying that farmers are concerned with public perception. The concern that producers have with consumers’ views of the dairy industry and farmers in general is important to note because it highlights the influence that individual dairy consumers have over the standards that they wish to see being upheld in the industry. Producer awareness of this group is critical and displays the impact that consumers can have on dairy farmers’ decisions to partake in certain calf care practices.

It is important to highlight the proportion of producers in this study who are not fully in compliance with proAction requirements for disbudding and dehorning, due to only using one of two required pain control medications for this practice. Although the vast majority of participants are in compliance with proAction requirements, nearly ¼ of producers are not highlighting the importance of additional factors that must be considered when understanding barriers to adoption of best management practices. While requirements and quality assurance programs may be an instigating factor for many producers, this appears not to be sufficient for a proportion of producers to adopt best practices for disbudding and dehorning and speaks to the limitations that may exist with programs such as this one.

### 4.5. Study Limitations

Several limitations exist when considering the results of this research study. This study was based on producer opinions and perspectives (specifically, the opinions of a sample of 29 producers from Ontario); therefore, the results of this research are limited in their ability to be extrapolated to wider populations; however, this is typically not the goal of qualitative research [19]. While the opinions and views of all Canadian farmers are not represented in this research, the information gained from the perspectives of these participants will provide further depth on this topic. The researchers feel that the data gained from this study support the findings of previous research and provide valuable information and insight into the barriers and perceptions surrounding pain control use for disbudding and dehorning. Developing a better understanding of how this group of Ontario producers feels about this practice is informative for the dairy science community, as it allows for stimulation of new hypotheses and ideas for future research surrounding factors that might motivate or inhibit producers from adopting pain control use for these practices. Limitations of interviews conducted over the phone include a lack of personal connection between the producer and the interviewer [40]; however, this method allowed the researcher and participant to abide by safety protocols and COVID-19 restrictions and encouraged honest participant responses by increasing the level of confidentiality.

Additional limitations to consider include those specific to qualitative research, such as the validity and reliability of this study. Qualitative data are highly subjective and is not intended to reach consensus among participants, which means that the robustness of the data cannot be evaluated in the same way as quantitative data [19,20]. Validity and reliability of qualitative research can be established through various measures, such as acknowledgement of biases through reflexivity, repeated examination of data, and participant validation [23,24]. All researchers followed well-established thematic analysis protocols and the researchers recognized their own biases when interpreting these data. Therefore, the researchers of this study feel that they have produced a robust study that accurately reflects the opinions and perspectives of the participants in this project.

## 5. Conclusions

In general, dairy producers’ attitudes were highly influential on their decision to use pain mitigation for disbudding and dehorning. Study participant attitudes surrounding pain control use and their willingness to use some form of pain mitigation for disbudding and dehorning suggests that these producers are concerned about the health and welfare of their calves; however, more comprehensive education of producers in general is needed to encourage full compliance with *proAction* requirements. Veterinarians were seen as influential sources for participants and were cited as a tool to remove barriers to pain control use. This suggests that veterinarians could be a beneficial educational source for producers, demonstrating their opportunity to encourage positive change towards the best disbudding and dehorning practices by producers.

## Figures and Tables

**Table 1:** A description of the disbudding and dehorning practices for 29 Ontario dairy farmers. This information is included for reference and to provide additional context to the reader.

Who Is the Primary Individual(s) Who Perform(s) the Disbudding or Dehorning on Your Farm? (*n* = 29)	Number of Participants
Interview participant	16
Veterinarian	9
Family and/or staff members	3
Participant disbuds/dehorns and veterinarian applies pain control	1

**Table 2:** A description of the medications used by 29 Ontario farmers for the disbudding and dehorning of dairy calves. This table is included for reference and to provide additional context to the reader. In total, an NSAID (meloxicam) was used by 26 participants, a local anaesthetic block (lidocaine) was used by 25 participants, and 13 participants used a sedative. Twenty-two participants were in full compliance with proAction requirements for disbudding (NSAID + local anaesthetic at minimum).

What Medications (If Any) Do You Use When Disbudding or Dehorning Your Calves? (*n* = 29)	Number of Participants
NSAID * + lidocaine + sedative	12
NSAID + lidocaine	10
NSAID	3
Lidocaine	3
NSAID + sedation	1

* Non-Steroidal Anti-Inflammatory Drug.

## Data Availability

The data presented in this study are available in this article.

## Appendix A. Phone Interview Discussion Guide

### Begin Recording

Introduction to Interview (approx. 5 minutes)

Hello and welcome.

My name is _____ and I will be guiding the conversation today. My role is to facilitate discussion, ask questions and remain unbiased to allow you to freely share your thoughts and opinions. As you have read in the Consent Document, we are conducting this study to better understand the opinions and perspectives of producers towards disbudding practices. You were asked to participate in this interview because of your role as a farmer or farm staff and I am interested in hearing your perspectives on:

- The use of pain control when disbudding/dehorning calves
- The current quality assurance programs in your country

Please try to speak clearly when answering questions, as this interview is being recorded for future analysis.

You will not be named or have your name associated with anything that is said during this interview. If you discuss any other individuals during this recording, please refrain from using identifiable information to keep them anonymous as well. If you do use names or other identifying information, know that it will be removed from the transcripts.

We may use verbatim quotes of things that are said during this interview in papers or reports that arise from this work; however, you will not be identified in any way. Please feel free to share all of your honest thoughts and opinions on the topics that we will discuss today and don’t worry about making statements that you think might be uncommon or not shared by myself. I am simply here to hear your opinions and experiences and will not pass judgement. Remember that you have the option of withdrawing from this study at any time or opting to skip any questions that make you uncomfortable. If you would like to leave the interview at any point, please just let me know.

Your gift card will be mailed to you following your interview. Are there any questions that you have before we continue on to the interview?

**Objective**: To understand the main barriers and/or incentives that are motivating producers’ decisions regarding pain control when disbudding calves.

Introduction Questions:

**Aim**: to better understand the producer and their farm.

1. Please tell me about your farm
  - What is the size of your herd?
  - Where is your farm located?
  - How long have you been farming?

Disbudding Protocol:

**Aim**: to understand what the producer views as the ideal disbudding protocol, their willingness to adapt to changing requirements and if they use “progressive” disbudding strategies.

2. Let’s talk a little bit about disbudding. Walk me through disbudding on your farm, what does your disbudding protocol look like?
  - At what age do you disbud?
    ○ Do you use medication?
  - Who does the disbudding on your farm?
3. How long have you used this disbudding protocol?
4. Why is the protocol that you are currently using your preferred method?
  - Do you find your current disbudding protocol to be successful?
  - Are there any challenges that you currently face with this disbudding protocol?
5. How did you develop the disbudding protocol that you currently use?
  - Did anyone assist you with protocol development?
  - Did you seek information from other sources?

Pain Medication:

**Aim**: to understand the real or perceived barriers that prevent producers from using pain control, their general perception of pain control, why they choose to use pain control and how they think their peers perceive pain control.

6. To talk more specifically about disbudding medications, what are your thoughts on using pain control drugs like meloxicam or lidocaine during disbudding?
  - Why?
7. Do you feel that other members of the dairy community share the same perspectives as you?
  - Why or why not?
  - Can you elaborate on that?
8. The 2015 National Dairy Study in Canada showed that around two thirds of producers are using pain control when they disbud or dehorn their calves. What are your thoughts on this?
  - Does this surprise you?
9. What do you think are the main reasons that producers would choose to use pain control?
  - Why?
10. Why do you think producers would **not** choose to use pain control?
  - What real or perceived barriers exist for them?
11. If all existing barriers were suddenly eliminated, do you think pain medication should be used when disbudding calves?
  - Why or why not?
12. Do you think it’s possible to eliminate all barriers for producers?
  - Why or why not?
  - How would these barriers be removed?
  - What would that look like to you?

Current Requirements

**Aim**: to understand what the producer thinks about the current requirements in place today and if there are ways that they can be adjusted to better support producers.

13. ProAction has a requirement for disbudding (since Sept 2019), which states that pain control in the form of an anaesthetic (freezing) and an NSAID at minimum, must be administered before dehorning. What are your thoughts on this?
  - Did you know about the requirements?
  - Do you think they are fair?
  - Are they too restrictive?
  - Should they be more specific?
  - What would you change if you could?
14. Do you think other producers have the same perspectives as you on this? Why or why not?
15. What is your opinion on ProAction in general?
  - Why?
  - What has it changed in terms of the way you do things on your farm?
16. What would you want to change about ProAction, with regards to disbudding requirements, or in general for the program?
17. That brings me to the end of my list of questions for you. Is there anything else you wanted to discuss or add before we wrap up?
  - Any questions for me or things you wanted to mention?

Summary:

This brings us to the end of the interview. I want to thank you for sharing your thoughts and opinions with me today. This will be very helpful in allowing us to better understand producers’ perspectives on disbudding and pain control. Once again, your responses will be kept confidential and any direct quotes that are used from this interview will be anonymized. Please let me know if you would like a copy of the transcript emailed to you. If you can think of anyone else who might be interested, please let me know. Your $25 Tim Horton’s gift card will be mailed to you this week. Is there an address that works best for me to send it to?

Thank you so much for your time!
